# Combined activity of COX-1 and COX-2 is increased in non-neoplastic colonic mucosa from colorectal neoplasia patients

**DOI:** 10.1186/s12876-018-0759-1

**Published:** 2018-02-27

**Authors:** Thorbjørn Søren Rønn Jensen, Badar Mahmood, Morten Bach Damm, Marie Balslev Backe, Mattias Salling Dahllöf, Steen Seier Poulsen, Mark Berner Hansen, Niels Bindslev

**Affiliations:** 1 Digestive Disease Center K, Bispebjerg Hospital, DK-2400 Copenhagen, NV Denmark; 2 Department of Biomedical Sciences, Faculty of Health Sciences, University of Copenhagen, DK-2200 Copenhagen N, Denmark

**Keywords:** Short circuit current (SCC), Cyclooxygenase, Endoscopic, Biopsy, Carcinogenesis, Tuft cells

## Abstract

**Background:**

Cyclooxygenase (COX) activity is increased in endoscopic normal colonic mucosa from patients with colorectal neoplasia (CRN). COX-2 is thought to be the predominant COX isozyme involved in neoplasia. Meanwhile, relative contributions of COX-1 and COX-2 isoforms are unknown. Knowledge about their mutual activity in colonic mucosa is important for diagnostics and targeted therapy for CRN. The aim of this study was to assess the relative function, expression and localization of COX-1 and COX-2 enzymes in colonic non-neoplastic human mucosa and thereby to potentially reveal a mucosal disease predisposition for better treatment.

**Methods:**

Biopsies were pinched from normal appearing colonic mucosa in patients undergoing endoscopy. Ussing chamber technique was applied for an indirect assessment of epithelial activity, RT-qPCR for expression and immunohistochemistry for localization of COX-1 and COX-2 enzymes in patients without (ctrls) and with a history of CRN (CRN-pts).

**Results:**

Combined COX-1 and COX-2 activity was higher in CRN-pts, *p* = 0.036. COX-2 was primarily localized in absorptive cells, while COX-1 appeared to be restricted to nonenteroendocrine tuft cells of the colonic epithelium.

**Conclusions:**

In biopsies from endoscopic normal appearing colonic mucosa, combined activity of COX-1 and COX-2 enzymes is increased in CRN-pts compared with ctrls. This indicates that COX-1 and COX-2 together contribute to an increased proliferation process. Of note, in colonic epithelial cell lining, the COX-1 enzyme seems localized in tuft cells.

## Background

In the Western world, colorectal cancer (CRC) caused some 694,000 deaths in 2012, making it the third most common type of cancer and the second leading cause of cancer-related death [1]. Observation and documentation of possible altered signaling in pre-neoplastic colorectal mucosa from humans are essentials for future development of targeted pharmacotherapy against CRC and colorectal neoplasia (CRN).

Epidemiological studies show that daily intake of non-steroidal anti-inflammatory drugs (NSAIDs) reduces long-term incidence of developing CRC [2, 3]. Based primarily on these data, the United States Preventive Services Task Force (USPSTF) recently recommended routine use of low-dose aspirin for chronic disease prophylaxis, including CRC prevention, among adults between ages 50 and 59 with a > 10% risk of cardiovascular events [4, 5].

The mechanism behind NSAIDs’s chemoprevention is most likely due to inhibition of cyclooxygenase (COX) enzymes, although other mechanisms of aspirins CRN-prevention are possible [6]. COX enzymes convert arachidonic acid into various metabolites including prostaglandin E2 (PGE_2_). PGE_2_ appears to be involved in neoplastic changes owing to its proinflammatory properties. Further, PGE_2_ has been demonstrated to promote proliferation, cell migration, angiogenesis and reduce apoptosis in colonic mucosal lining [7, 8]. The COX enzyme exists in two major and clinically relevant isoforms: COX-1 and COX-2. COX-1 is known to have constitutive activity, while COX-2 is an infectious and injury-inducible enzyme.

Our research group has previously identified up-regulated indomethacin-sensitive COX activity in biopsies taken from endoscopically normal appearing colonic mucosa when compared between patients with a history of or present CRN to controls [9]. Another study by us showed a significantly augmented expression of two potential PGE_2_ inward transporters, OATP2B1 and OATP4A1, in colonic biopsies from patients with CRN compared to patients without CRN. This up-regulated expression points to a compensatory increased basolateral PGE_2_ uptake into colonic columnar epithelial cells associated with neoplasia [10]. This study also suggested the ABCC5 transporter as the epithelial cell PGE_2_ exporter rather than the ABCC4 transporter.

The importance of COX enzyme subtypes for development of CRC is generally assumed to be due to an increased activity of the COX-2 isozyme [11]. However, this view is mostly based on studies on transformed cells or malignant tissue samples. It is well-known that the expression and activity of effector molecules, such as enzymes, transporters and receptors, often are dramatically altered in cell lines and in cancerous tissues as a byproduct of the cancer process itself. So far, to our knowledge, the relative contribution of COX-1 and COX-2 subtypes to an up-regulated COX activity, causing CRN, has not yet been fully elucidated. Accordingly, we decided to determine possible altered activity of both COX-1 and COX-2 in endoscopically normal appearing colonic mucosa from individuals with CRN when compared to controls. Choosing a comparison between normal appearing mucosa from both patient groups, further give a potential opportunity to evaluate if the colonic mucosa in patients with CRN is predisposed for development of the disease.

**Fig. 1:**
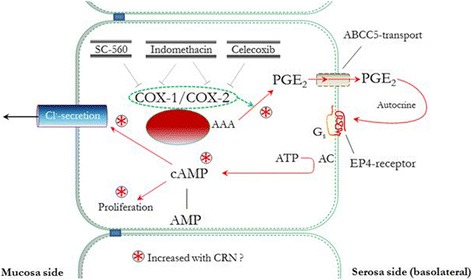
A simplified scheme for involvement of COX enzymes in development of CRN. COX-1 and COX-2 convert active arachidonic acid (AAA) into PGE_2_, which at the basolateral side is transported out off the cell via an ABCC5-transporter. Through a Gs-protein-coupled EP-4-receptor, PGE_2_ mediates conversion of ATP to cAMP, thus inducing mucosal Cl^−^ secretion and cell proliferation. COX isozymes may be inhibited selectively by SC-560 and celecoxib or non-selectively by indomethacin. Pathways with  markers are hypothetically increased in CRN, thereby increasing cell proliferation and carcinogenesis. PGE_2_: prostaglandin E2, cAMP: cyclic adenosine monophosphate, AMP: adenosine monophosphate, ATP: adenosine triphosphate, COX: cyclooxygenase, CRN: colorectal neoplasia, G_s_: stimulatory heterotrimeric G protein, ABCC5: ATP-binding cassette transporter C5, EP4: prostaglandin receptor subtype 4, SC-560: selective COX-1 inhibitor, celecoxib: selective COX-2 inhibitor

We hypothesized that activity of both COX isozymes might be up-regulated even in normal appearing mucosa and thereby together contribute to development of CRN, Fig. 1.

Accordingly, the aim of this study was to assess the relative functional importance, expression and localization of COX-1 and COX-2 enzymes in endoscopically normal appearing colonic mucosa from patients without (ctrls) and with colorectal neoplasia (CRN-pts), and furthermore to potentially reveal a mucosal predisposition for the disease.

## Methods

### Study population

Adult patients (≥ 18 years), referred for colonoscopy, were invited to participate. Patients were pooled into the neoplasia group if they presented a history of CRN. Patients with no present endoscopic signs or history of CRN served as ctrls. Patients with hemorrhagic diathesis or inflammatory bowel disease were excluded from the study. For each patient, we noted medication, body mass index (BMI), previous illnesses, all signs of earlier colorectal disease and the findings during the colonoscopy.

Forty three patients were enrolled in the study (19 women). For Ussing chamber studies, biopsies from randomly selected 22 CRN-pts (10 women) and 21 ctrls (9 women) were examined. In the real time polymerase chain reaction study (RT-qPCR), biopsies from randomly selected 11 CRN-pts (6 women) and 7 ctrls (4 women) were included. For immunohistochemistry, biopsies from randomly selected 8 CRN-pts (3 women) and 7 ctrls (3 women) were assessed. Eleven patients, both CRN-pts (2 women, 3 men) and ctrls (3 women, 3 men), had comorbidity such as diabetes, hypertension, atrial fibrillation, prostatic cancer, chronic obstructive pulmonary disease and dyslipidemia.

The study protocol was approved by the scientific Ethical Committee of Copenhagen (H-3-2013-107) and the Danish Data Protection Agency approved the study protocol (BBH-2013-024, I-suite no: 02342). The study was conducted in accordance with the Helsinki declaration. Patient information and data were collected and stored in locked containers.

### Statistical analysis

Mann-Whitney Rank Sum test or unpaired t-test was used for the calculation of *p*-values depending on the results of normality and equal variance tests. *P*-values less than 0.05 were considered significant. All statistics were done with SigmaPlot 12.3 for Windows (Systat Software Inc., USA/Canada). Data are presented as mean (±SEM).

### Chemicals

Theophylline, indomethacin, prostaglandin E2 (PGE_2_), SC-560 and celecoxib were purchased from Sigma-Aldrich (Seelze, Germany). Amiloride, bumetanide and ouabain were purchased from Sigma (Vallensbaek Strand, Denmark).

The antibody for COX-2 (cat. no.: SB-M3210) was obtained from Nordic BioSite ApS (Täby, Sweden) and antibodies for COX-1 from Santa Cruz Biotechnology (cat. no.: sc-1752, sc-7950, and sc-19998; Santa Cruz, CA, USA) and Abcam Cambridge (cat. no.: ab109025; Cambridge, UK). Fluorescence double labeling was conducted with antibodies for the following entities: serotonin (cat. no.: M075801-2) and chromogranin A (cat. no.: M086929-2) were purchased from Dako (Glostrup, Denmark); proprotein convertase-1 and -2 (PC1 and PC2; cat. no.: alx-210-518-R100 and alx-210-519-R100) from Enzo Life Sciences (Varazdin, Croatia); somatostatin (cat. no.: sc-13099) from Santa Cruz Biotechnology (Santa Cruz, CA, USA); gastric inhibitory polypeptide (GIP, cat. no.: ABS021-04-02) from Thermo Scientific (Rockford, USA) and glucagon-like peptide-1 (GLP-1; cat. no.: 87805-34) from Tocris Bioscience (Bristol, UK).

Primer sequences were synthesized by TAG Copenhagen (Copenhagen, Denmark). All other chemicals were of analytical grade.

### Biopsy procedure

Six biopsies were obtained from each patient. Of the six biopsies, one was used for RT-qPCR, one for immunohistochemistry and four biopsies were mounted in modified air-suction Ussing chambers [9]. During endoscopy, biopsies were pinched from normal appearing mucosa approximately 30 cm orally from the anal verge and at least 10 cm from abnormal tissue on retraction of the endoscope. Standard biopsy forceps (Boston Scientific, Radial Jaw 4, outside diameter of 2.2 mm) were used. Biopsies were placed in iced Ringer-solution and immediately transferred to the laboratory for mounting in Ussing chambers.

Three experimental techniques were employed:

Ussing chamber (A), RT-qPCR (B) and immunohistochemistry (C)

A. *Functional studies*

Biopsies were mounted within 30 min in Ussing chambers [9]. Mounting was carried out at 10 times magnification by means of a stereomicroscope to secure correct mucosa-serosa orientation and proper fixation. Both sides of the tissue were bathed in bicarbonate-Ringer solution containing (in mM) 140 Na^+^, 4 K^+^, 121 Cl^−^, 1 Ca^2+^, 0.5 Mg^2+^, 0.5 SO_4_^2−^, 25 HCO_3_^−^, and 5.5 D-glucose. Solutions were oxygenated with 95% O_2_/5% CO_2_, i.e., buffered to pH 7.4, and circulated by gas-lifts. Temperature was maintained at 37 °C by water jackets. Short circuit current (SCC, μA·cm^− 2^) and slope conductance (G, mS·cm^− 2^) were recorded continuously using an automated voltage-clamp device. Correction for solution resistance was performed immediately before specimens were mounted. The slope conductance was only used as a control for acceptable slit fixation ranging between 60 and 120 mS per sq. cm.

Experiments were initiated following a minimum equilibration period of 10 min. Amiloride (20 μM, apical side) was added to inhibit sodium channels (ENaCs). Theophylline (400 μM, both sides) was then added to raise the level of cAMP due to inhibition of phosphodiesterase activity and thus optimizing the effect of COX subtype inhibitors. When the SCC was stable, a selective inhibitor of either COX-1 (SC-560, 500 nM, both sides) or COX-2 (celecoxib, 500 nM, both sides) was added. After 30 min or when the SCC reached a plateau, indomethacin was added (13 μM, both sides). Again, after 30 min or when SCC had stabilized, PGE_2_ (100 nM, serosal side) was added. Finally, at the end of the experiment, bumetanide (25 μM, serosal side) was added as a measure for induced chloride secretion and followed by ouabain (200 μM, serosal side) as a control of biopsy viability. Selection of half-chamber concentrations for the various drugs was based on pharmacodynamic experience from previous studies.

B. *Expression studies*

From each patient included here, one biopsy was obtained and immediately transferred to RNAlater (Life Technologies, Naerum, Denmark). Biopsies were homogenized using a TissueLyser II (Qiagen, Copenhagen, Denmark), and subsequently RNA was extracted using NucleoSpin RNA® (Macherey-Nagel, Düren, Germany). Concentration and purity of RNA were determined using a NanoDrop® ND-1000 (NanoDrop Technologies, Wilmington, DE, USA), the latter by the A_260_/A_280_ and A_260_/A_230_ absorbance ratios. RNA was converted to cDNA using the iScript™ cDNA Synthesis Kit (BioRad, Copenhagen, Denmark) according to the manufacturer’s protocol. Primers against genes of interest and ß-actin were designed using Primer3 (http://frodo.wi.mit.edu/primer3/input.htm) based on sequences obtained from Ensembl (www.ensembl.org). The primer sequences were synthesized by TAG Copenhagen (Copenhagen, Denmark): COX-1 forward (5’-GAGCAGCTTTTCCAGACGA -3′); COX-1 reverse (5′- TCCTCGATGACAATCTTGATG -3′); COX-2 forward (5′- ACTAGAGCCCTTCCTCCTGTG -3′); COX-2 reverse (5′- GGGATCAGGGATGAACTTTCT -3′); ß-Actin forward (5’-ACCCAGCACAATGAAGATCA-3′); ß-Actin reverse (5’-CGTCATACTCCTGCTTGCTG-3′). Dilution series of cDNA from HEK293 cells were run to verify acceptable amplification efficiencies and specificities by standard and dissociation curves for all primer sets. cDNA was amplified on a 7900HT Fast Real-Time PCR System (Applied Biosystems, Foster City, CA, USA) using Fast SYBR® Green Master Mix (Applied Biosystems) in accordance with the manufacturer’s manual. Samples were run in triplicates with ß-actin primers as reference gene on all plates. Results were analyzed using SDS 2.3 (Applied Biosystems), and expression was calculated by the 2^-ΔCT^ method.

C. *Localization and abundance studies*

Immunohistochemical staining was performed to localize and quantify the two COX isoforms. One colonic biopsy from each patient, included for this part, was put aside in 4% neutral buffered formaldehyde right after the endoscopy procedure. Biopsies were subsequently embedded in paraffin and cut in 4 μm thin slices. The sections were deparaffinated and rehydrated, followed by heat treatment in a microwave oven in order to unmask epitopes. The sections were blocked with a 2% bovine serum albumin solution for 10 min to rule out unspecific antibody adhesion, followed by incubation with a primary antibody at 4 °C overnight. Images were recorded using a Zeiss Axioplan 2 plus microscope (Jena, Germany) fitted with a Photometrics CoolSNAP camera (Tucson, AZ, USA) and analysis was performed using Image-Pro Plus 7.0 software.

Immunohistochemical staining of COX-1 turned out insufficient for three of the antibodies and a fourth (sc-1752) stained single, open enteroendocrine-like cells in the epithelium, Fig. 3. To verify that the coloring was of non-enteroendocrine cells, double labeling immunofluorescence with sc-1752 and various markers for endocrine cells was performed. The following antibody concentrations were used: COX-1 (sc-1752) 1:800, COX-2 1:400, GLP-1 1:375, serotonin 1:100, somatostatin 1:2500, PC1 1:1250, PC2 1:1600, chromogranin A 1:10000 and GIP 1:1750.

For COX-2, all biopsies were quantified by a blinded investigator. Quantification images were recorded at 20× magnification and the area measured represented 186,000 μm^2^ of the tissue. The area of stained structures was quantified by selecting a colored region of interest. Automatically, areas with same color were measured. One image from each biopsy was measured. Blinded quantification of COX-2 was repeated 3 times. Data were calculated as mean area μm^2^ ± SEM for each group. Images for localization were recorded using a Zeiss Axio10 Imager A1 microscope (Jena, Germany) fitted with a Zeiss AxioCam ICc 3 camera (Jena, Germany) and analysis was performed using Image-Pro 9.1 software. Only mucosal layers were analyzed.

## Results

### Study population

We found no differences in comorbidity or in medication between the two patient groups. BMI and age was higher in CRN-pts compared to ctrls with BMI displaying statistically significance (BMI: CRN-pts 27.2 (±1.2) vs ctrls 23.2 (±1.2), *p* = 0.048. Age: CRN-pts 69.5 (±4.1) years vs ctrls 58.4 (±5.4) years, *p* = 0.062).

A. *Function*

Baseline SCC and differential effects of amiloride, theophylline, indomethacin, PGE_2_, bumetanide and ouabain on SCC are listed in Table 1. Inhibition of SCC by amiloride was more pronounced in CRN-pts compared to ctrls, *p* = 0.006. Stimulation of SCC by theophylline was significantly more efficacious in CRN-pts compared to ctrls, *p* = 0.025.

**Table 1:** Drug-induced changes in short circuit current (SCC)

	CRN-pts Mean ΔSCC, SEM (μA·cm^− 2^)	CRN-pts N/n	Ctrls Mean ΔSCC, SEM (μA·cm^− 2^)	Ctrls N/n	*p*-value
Baseline SCC	91 ± 10.1	22/42	95 ± 26.6	21/30	0.518
Amiloride	−77 ± 13.4	17/38	−30 ± 15.1	13/22	0.006 *
Theophylline	73.5 ± 7.1	17/38	50.5 ± 6.9	13/22	0.025 *
SC-560 + Celecoxib	−66.7 ± 3.5	17/38	−54.7 ± 4.3	13/22	0.036 *
PGE_2_	87.5 ± 32.3	15/26	73.0 ± 17.3	10/19	0.275
Bumetanide	−41.5 ± 5.3	15/32	−55.0 ± 9.4	7/18	0.261
Ouabain	−70.5 ± 14.4	13/24	−93.0 ± 14.7	7/18	0.431

Baseline SCCs are absolute values, while amiloride (20 μM, apical), theophylline (400 μM, both sides), SC-560 + Celecoxib (500 μM, both sides), prostaglandin (PGE_2_) (100 nM, serosal), bumetanide (13 μM, serosal) and ouabain (200 μM, serosal) effects are changes from prestimulatory SCC (ΔSCC). SC-560 and Celecoxib are selective COX-1 and COX-2 inhibitors. SC-560 + Celecoxib represent the combined SCC inhibition data of both COX-1/indomethacin and COX-2/indomethacin application. CRN-pts values represent SCC or ΔSCC in biopsies from colorectal neoplasia patients and ctrls values are for patients without colorectal neoplasia. *N* = number of patients, *n* = number of biopsies, in parenthesis (N/n). * *p*-value < 0.05

**Fig. 2:**
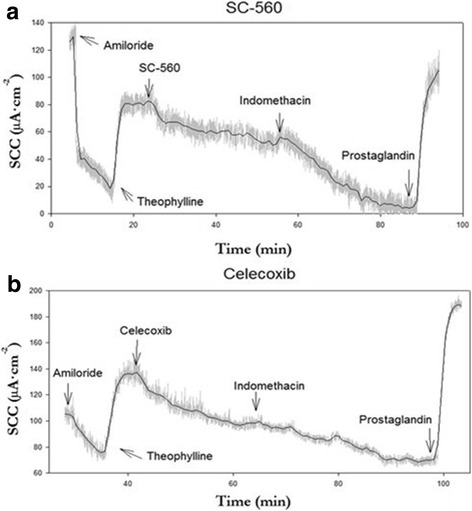
Examples of typical recording in Ussing Chamber experiments on short circuit current (SCC) following exposure to COX-1 (cyclooxygenase) inhibitor SC-560 (**a**) and COX-2 inhibitor celecoxib (**b**). Compounds were added to biopsies in the following concentrations: amiloride (20 μM, mucosal side), theophylline (400 μM, both sides), either COX-1 inhibitor (SC-560, 500 nM, both sides) or COX-2 inhibitor (celecoxib, 500 nM, both sides), indomethacin (13 μM, both sides) and prostaglandin (PGE_2_, 100 nM, serosal side)

Total SCC response to COX inhibition was calculated by adding the response of either selective inhibitor to the ensuing response of indomethacin. The decrease in SCC to either of the selective COX-inhibitors was normalized based on total COX inhibition for the individual biopsy. Examples of Ussing chamber experiments of inhibition of either COX subtype can be seen in Fig. 2. No difference in SCC inhibition between patients group was observed for either of the two selective COX inhibitors. By contrast, the SCC response of combined COX-1 and COX-2 inhibition, after indomethacin application, was significantly larger in CRN-pts compared to ctrls, *p* = 0.036, Table 1. This significant data difference corroborates a significantly larger drop in SCC to indomethacin found in a previous study for CRN-pts compared to ctrls [9].

When comparing the fractional contribution of COX-1 and COX-2, both normalized with total COX activity, COX-1 contribution was significantly higher compared to COX-2 both for ctrls (*p* = 0.028) and CRN-pts (*p* = 0.035), Table 2.

B. *Expression*

**Table 2:** Percentage drop in total COX-induced short circuit current by selective inhibitors, COX-1 (SC-560) and COX-2 (Celecoxib)

	SC-560 Inhibition, SEM (%)	Celecoxib Inhibition, SEM (%)	*p*-value
CRN-pts (N/*n* = 15/26)	68.8 ± 4.5	53.7 ± 5.1	0.035*
Ctrls (N/*n* = 10/19)	80.6 ± 4.8	52.6 ± 10.3	0.028*
*p*-value	0.10	0.91	

Horizontal *p*-values to the right show inhibition by SC-560 compared to inhibition by Celecoxib for CRN-pts and ctrls. Vertical *p*-values at the bottom show CRN-pts compared to ctrls for SC-560 inhibition and Celecoxib inhibition. Inhibition calculated as percentage selective COX-inhibition of total COX-inhibition. Total COX-inhibition defined as selective COX-inhibition + indomethacin inhibition. Percentage drop reflects the overall decreased activity. CRN-pts represent values for biopsies from colorectal neoplasia patients and ctrls values are for biopsies from patients without colorectal neoplasia. *N* = number of patients, *n* = number of biopsies, in parenthesis (N/n). * *p*-value < 0.05

Using RT-qPCR, we examined the expression of COX-1 and COX-2 in colonic mucosa from CRN-pts and ctrls. We further wanted to compare the combined expression of COX-1 and COX-2 between the two patient groups.

The expression of COX-1 was significantly higher compared to COX-2 both in CRN-pts (COX-1 = 0.024 (±0.003) vs COX-2 = 0.007 (±0.002), *p* = 0.012) and in ctrls (COX-1 = 0.020 (±0.003) vs COX-2 = 0.005 (±0.002), *p* < 0.001). Comparing CRN-pts with ctrls, mRNA-expression of both COX-1 and COX-2 was numerically higher in CRN-pts, although upregulation of both genes failed to reach statistical significance (COX-1: *p* = 0.249, COX-2: *p* = 0.431), Table 3. Combined expression of the COX isozymes was defined as the summed expression of COX-1 and COX-2. Comparing combined COX expression for CRN-pts and ctrls showed no statistical significant difference (CRN-pts = 0.015 (±0.003) vs ctrls = 0.012 (±0.003), *p* = 0.353).

C. *Localization and abundance*

**Table 3:** COX-1 and COX-2 expression based on real time polymerase chain reaction in colonic biopsies from CRN-pts and ctrls

	COX-1 expression, SEM	COX-2 expression, SEM	*p*-value
CRN-pts (N/*n* = 11/11)	0.024 ± 0.003	0.007 ± 0.002	0.012 *
Ctrls (N/*n* = 7/7)	0.020 ± 0.003	0.005 ± 0.002	< 0.001 *
*p*-value	0.24	0.43	

Horizontal *p*-values to the right compare COX-1 expression with COX-2 expression for CRN-pts and ctrls. Vertical *p*-values at the bottom compare CRN-pts with ctrls for COX-1 and COX-2 expression. Total or combined expression of COX enzyme is defined as expression of COX-1 + COX-2. The combined COX expression was 0.015 ± 0.003 for CRN-pts and 0.012 ± 0.003 for ctrls; *p* = 0.35. CRN-pts represent values for biopsies from colorectal neoplasia patients and ctrls values are for biopsies from patients without colorectal neoplasia. *N* = number of patients, *n* = number of biopsies, in parenthesis (N/n). * *p*-value < 0.05

**Fig. 3:**
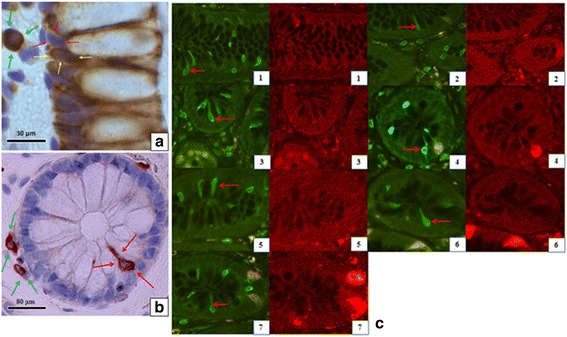
Immunohistochemical staining of colonic mucosa with cyclooxygenase (COX) subtype specific antibodies in patients with colorectal neoplasia. **a** COX-2 immunohistochemical staining appears localized to the cytoplasm of absorptive cells (yellow arrows). No staining is seen in goblet cells (red arrows). A pericryptal stromal cell is marked (green arrow). **b** COX-1 immunohistochemical staining, here with antibody sc-1752, appears to stain morphological appearing tuft cells (red arrows) and in the lamina propria proposedly myofibroblasts (green arrow). **c** Fluorescence double labeling shows no co-localization between COX-1 (green) and specific markers for endocrine cells (red). Arrows point at COX-1 positive tuft cells. Subfigures 1, 2, 3, 5 and 7 have proportions of 100 μm per centimeter, subfigures 4 and 6 have proportions of 50 μm per centimeter. 1: Glucagon-like peptide-1, 2: Somatostatin, 3: Gastric Inhibitory Polypeptide, 4: Proprotein Convertase-1, 5: Proprotein Convertase-2, 6: Serotonin, 7: Chromogranin A

Localization of COX-2 was immunohistochemically delimited to cytoplasm of absorptive cells, Fig. 3a. Quantification of COX-2 showed no significant difference between CRN-pts and ctrls, CRN-pts: 1472 ± 168 μm^2^ and ctrls: 1398 ± 132 μm^2^, *p* = 0.362.

Since neither of the classical makers for endocrine cells co-stained with sc-1752 for the COX-1 enzyme, we conclude that the sc-1752 antibody marks tufts cells in the epithelial lining as also reported by Gerbe et al. ([12], and their supplemental material Fig. S1A). Use of two other antibodies gave either smeared stains or no staining.

Double fluorescence staining was made with COX-1 antibody sc-1752 and antibodies for the following endocrine cell-markers: chromogranin A, GIP, serotonin, GLP-1, somatostatin, PC1 and PC2. None of the markers for endocrine cells were found to co-localize with COX-1, see Fig. 3c, thus confirming the coloring of non-enteroendocrine epithelial cell, identified as tuft cells, and also of unspecified solitary stromal cells. In a preliminary enhanced fluorescence immuno-histochemical study of COX-1 in human colonic biopsies in our laboratory, there is a clear subepithelial localization of the protein in solitary cells with a morphology resembling myofibroblasts (Dr. Hanne Borger Rasmussen and Christian Hunnicke Petersen, unpublished results).

## Discussion

Our study seeks to evaluate parameters related to the content and impact of PGE_2_ in normal human colonic mucosa. We chose to study normal appearing colonic mucosa from ctrls and CRN-pts in order to try to evaluate a possible predisposition for the disease in normal tissue from CRN-pts when compared with ctrls. Thus, in biopsies from this mucosa, we looked at a functional parameter, indomethacin-sensitive short circuit current (IS-SCC) in the presence of amiloride, including COX enzyme subtype-specific SCC inhibition. Although IS-SCC is indirectly related to the effects of PGE_2_, it is well-established as a measure of PGE_2_ activity working through a COX enzyme-adenylate-cyclase-Gs-cAMP pathway, Fig. 1. There are several advantages of IS-SCC, as a measure of functionality. Thus, IS-SCC is solely related to the epithelial cell layer and with superior sensitivity as compared for instance with enzymatic assays for the content of PGE_2_ and/or signaling pathway entities, most often determined for the entire mucosa, with low accuracy and thus limited by indirect congruency with epithelial functionality.

### COX-1 and COX-2 isozymes

A previous functional study from our laboratory reported an increased total COX activity in endoscopic normal appearing mucosa from patients with diagnosis of CRN [9]. The present study supports this observation, since the combined activity of COX-1 and COX-2 in colonic mucosa is significantly increased with CRN and cancer, though neither COX-1 nor COX-2 alone showed a significantly augmented activity.

Up-regulation of COX-2 is associated with increased cell adhesion, phenotypic changes, resistance to apoptosis and tumor angiogenesis [13–17]. These changes are possibly due to a raised prostaglandin E2 production, a notion supported by two laboratories, where Eberhart et al. found levels of prostaglandin E2 to be 3-4 fold increased in CRC tissue [18, 19]. PGE_2_ is known to inhibit apoptosis, to stimulate both tumor growth and angiogenesis and to act as an immunosuppressant in patients with CRC [13, 20, 21]. Even though COX-2 is a well-known enhancer of carcinogenesis in CRC, it has been proposed that both COX-1 and COX-2 pathways are involved in intestinal tumorigenesis [17]. This statement is strongly supported by experimental animal studies as the loss of either COX-1 or COX-2 genes blocks intestinal polyposis in mouse models of familial adenomatous polyposis by approximately 90% [22, 23].

Knowledge about overexpression of various enzymes in neoplastic tissue is well established, but our study additionally indicates that NSAID-sensitive mechanisms are over-activate not only in neoplastic tissue, but also in endoscopic normal appearing mucosa in CRN-pts, thereby representing tissue with an predisposed potential condition for neoplastic development.

In the present study, expressional levels of constitutive COX-1 were statistically higher compared to COX-2 in both CRN-pts and ctrls, Table 3 horizontal *p*-values. This finding may be contrasted by considerations about the characteristics of the two isozymes. COX-1 gene is considered the “housekeeping enzyme” and is highly and constitutively expressed in platelets and in gastric epithelial cells. In the latter, it helps in cytoprotection through the generation of prostanoids [24, 25]. In contrast, the COX-2 enzyme is inducible by many factors: e.g., bacterial endotoxins, cytokines and growth factors. Accordingly, the COX-2 activity is typically transient [26]. Of note, this differentiation between the cyclooxygenase isozymes, more than often turns out to be too simplistic [27, 28].

### COX-1 detection and tuft cells

One of three employed COX-1 antibodies stained the biopsy mucosa specifically in sporadic epithelial cells. By double-staining for COX-1 and enteroendocrine cells (EECs), we could exclude a labeling of EECs, Fig. 3c. Therefore, we rationalized that the COX-1 staining detected tuft cells as also concluded in several mouse colon studies with COX-1 immunostaining [12, 29–31]. These cells (also known as brush cells) have previously been found in endoderm-derived epithelia [12]. Tuft cells have long and blunt microvilli with a protruding root system along a well-built tubulovesicular system in the cytoplasm near the nucleus [32]. Fairly recently, tuft cells have been acknowledged as an important part of the intestinal lining with an immunological role to play in helminthic infections [33], while a more strict combination of specific tuft cell markers is still debated [12, 29, 33, 34]. Functional studies of tuft cells related to human colon cancer are still scarce [35], while colonic tuft cells appear related to the defense against worm infections [12]. To conclude, the COX-1-dependent PGE_2_ production in human colonic mucosa seems likely derived solely from epithelial tuft cells and probably as well supplied from subepithelial immune cells, which also stained with COX-1 antibody, data not shown. This proposal and a quantification of tuft cells in CRN-pts compared with ctrls, requires a separate study.

PGE_2_-producing stromal cells, colored by COX-1 and COX-2 antibodies in Fig. 3a, b, and c, are possibly myofibroblast, as suggested by Powell et al. [36], immune cells, mesenchymal stem cells and other non-identified stromal cells. As mentioned in the Result section, there is also an expression of the COX-1 protein in solitary subepithelial myofibroblast-like cells of human colonic mucosa, although with a much fainter coloring than for other stromal cells and epithelial tuft cells shown in Fig. 3b and c. This finding of COX-1 in subepithelial myofibroblasts is in line with earlier findings in the mouse small intestine and of myofibroblasts cultured from a mucosal biopsy of human neonatal colon [36, 37]. Of note, some of the produced PGE_2_ in human colonic mucosa is most likely also produced by stromal myofibroblasts as demonstrated for cultured colonic myofibroblasts from mice [38].

### Study population

Due to differences in the basic characteristics of our two patient groups, our study presents certain potential limitations. BMI and age were apparently slightly higher in the CRN study population. These observations are in line with obesity and increasing age being known risk factors for developing CRN and CRC [39]. Furthermore, SCC inhibition with amiloride and stimulation with theophylline differed between the study groups, Table 1. The colonic SCC response to amiloride is known for its variability and dependence on food ingredients, e.g., salt intake [40, 41]. The observed difference in SCC induced by amiloride can therefore be an unrelated variation in salt intake in our human study cohort and needs to be clarified in future studies. The characteristic of a significantly larger stimulatory effect of theophylline in CRN-pts is currently under study in our laboratory with results indicating lower SCC response to phosphodiesterase 4, PDE4, inhibition in colonic mucosa from CRN-pts. The increased theophylline response found in CRN-pts in this study, Table 1, could therefore also be a result of compensatory elevated phosphodiesterase activity in patients with CRN [42].

### Perspective on the use of aspirin and other NSAIDs

In the last decade, the mechanism of daily low-dose aspirin as a CRN-preventive measure has been questioned. Originally, the benefits of low-dose aspirin intake were thought primarily due to its COX enzyme inhibitor effects [2, 43]. Also, the recently published USPSTF’s recommendation for aspirin use in the prevention of CRN and CRC has not yet reached a general endorsement, as additional groupings, conditions and more evidence are still needed for a broad acceptance [5]. Our study here adds experimental human data, which support medication with non-selective COX-inhibitors for the prevention and/or treatment of CRN and CRC.

The subject of aspirin supplementation is now further complicated by recent realizations about the mechanisms behind beneficial effects of aspirin treatment involving integrated and intracellular multi-signaling pathways, such as pathological Wnt-beta-catenin and MAPKinase signaling as well as dysregulated non-coding long-RNA epigenetics and platelet functions [3, 6, 44].

Notwithstanding, a supposed major CRC prevention mechanism is still the inhibition of COX enzymes elicited by aspirin and other NSAID drugs. The level of mucosal PGE_2_ is lifted with CRN due to increased COX activity and possibly also reduced catabolism by not of a 15-hydroxy-prostaglandin-dehydrogenase PGE_2_ break-down [31]. NSAID-inhibition in effect reduces a lifted PGE_2_ level presumed to drive an immunosuppression or immune evasion, that establishes an oncogenic milieu [45].

Therefore, it is still important to study the PGE_2_ metabolism, including steps of its synthesis and catabolism, as well as its pertinent signaling pathways. And also, bring these studies from the culture dish and animal cage to human individuals for a comparison between these PGE_2_ parameters obtained from normal colonic mucosa of affected patients and controls. The research presented here is such a study and has revealed an equal importance of the two COX enzyme subtypes, COX-1 and COX-2, for the development of CRN, as the combined COX-1 and COX-2 displayed higher activity in CRN-pts. As the study is a pilot study with few participants, it certainly warrants confirmation by similar studies with much larger cohort numbers.

Full consensus for the use of daily low-dose aspirin to prevent CRC development, in the USPSTF recommendation, is still limited by uncertainties about the balance between advantageous and adverse effects for prophylactic use of NSAID chemo-preventive medicine against colorectal neoplasia [5]. This is due to variable setups and outcomes of several large clinical trials together involving more than 70,000 test persons. Furthermore, in this context it is still a hope to obtain better individualized medication, precision medicine, and molecular bio-markers for a more accurate assessment of risk stratification [46].

There is an ongoing dramatic development in studies on the COX-2 downstream-enzyme mPGES-1 (microsomal prostaglandin E2 synthase 1) with associated research for its selective and clinically relevant inhibitors. Discovery of such mPGES-1 inhibitors are intensely chased for the prevention of COX-2 dependent PGE 2 production and their installment as therapeutics [47]. While we wait for clinically approved mPGES-1 inhibitors for CRN, our study rather point to a possible use of low-dose non-specific COX inhibitors. Other alternative approach for treating CRN and CRC involving PGE_2_ may come from the bourgeoning studies on the gut microbiota, where the microorganism *Fusobacterium nucleatum* is linked with development of CRC and suspected of inducing microRNA-21 to increase the levels of IL-10 and prostaglandin E2 [48, 49].

## Conclusions

We find that COX-1 and COX-2 jointly contribute to COX-overactivity in colonic mucosa from patients with colorectal neoplasia. The clinical implications of the study are important for possible medical treatment of colorectal neoplasia with COX inhibitors, as it points to the use of non-selective COX inhibitors rather than specific COX-2 inhibitors. Immunohistochemically, COX-2 localizes to the cytoplasm of absorptive cells, while cells morphologically appearing like endocrine cells, non-identifiable with ordinary endocrine cell markers, seem to be COX-1 positive. However, for the normal epithelium of human colon, confirmation of COX-1 enzyme localization needs additional studies.

## Abbreviations

BMI: Body mass index
COX: Cyclooxygenase
CRC: Colorectal cancer
CRN: Colorectal neoplasia
CRN-pts: Colorectal neoplasia patients
Ctrls: Patients without colorectal neoplasia
EECs: Enteroendocrine cells
ENaCs: Epithelial sodium channel
GIP: Gastric inhibitory polypeptide
GLP-1: Glucagon-like peptide-1
IS-SCC: Indomethacin-sensitive short circuit current
mPGES-1: mincrosomal prostaglandin E2 synthase 1
NSAID: Non-steroid anti-inflammatory drug
PC: Proprotein convertase
PGE_2_: Prostaglandin E_2_
RT-qPCR: Real time polymerase chain reaction
SCC: Short circuit current
USPSTF: United States Preventive Services Task Force

## References

[1] Siegel RL, Miller KD, Jemal A (2016). Cancer statistics, 2016. CA Cancer J Clin.

[2] Thun MJ, Namboodiri MM, Heath CW (1991). Aspirin use and reduced risk of fatal colon cancer. N Engl J Med.

[3] Drew DA, Cao Y, Chan AT (2016). Aspirin and colorectal cancer: the promise of precision chemoprevention. Nat Rev Cancer.

[4] Chan AT, Ogino S, Fuchs CS (2007). Aspirin and the risk of colorectal cancer in relation to the expression of COX-2. N Engl J Med.

[5] Chan AT, Ladabaum U (2016). Where do we stand with aspirin for the prevention of colorectal cancer? The USPSTF recommendations. Gastroenterology.

[6] Guo H, Liu J, Ben Q, Qu Y, Li M, Wang Y (2016). The aspirin-induced long non-coding RNA OLA1P2 blocks phosphorylated STAT3 homodimer formation. Genome Biol.

[7] Stack E, DuBois RN (2001). Role of cyclooxygenase inhibitors for the prevention of colorectal cancer. Gastroenterol Clin N Am.

[8] Chell S, Kaidi A, Kadi A, Williams AC, Paraskeva C (2006). Mediators of PGE2 synthesis and signalling downstream of COX-2 represent potential targets for the prevention/treatment of colorectal cancer. Biochim Biophys Acta.

[9] Kaltoft N, Tilotta MC, Witte A-B, Osbak PS, Poulsen SS, Bindslev N (2010). Prostaglandin E2-induced colonic secretion in patients with and without colorectal neoplasia. BMC Gastroenterol.

[10] Kleberg K, Jensen GM, Christensen DP, Lundh M, Grunnet LG, Knuhtsen S (2012). Transporter function and cyclic AMP turnover in normal colonic mucosa from patients with and without colorectal neoplasia. BMC Gastroenterol.

[11] Ahmed M, Hussain AR, Siraj AK, Uddin S, Al-Sanea N, Al-Dayel F (2015). Co-targeting of Cyclooxygenase-2 and FoxM1 is a viable strategy in inducing anticancer effects in colorectal cancer cells. Mol Cancer.

[12] Gerbe F, van Es JH, Makrini L, Brulin B, Mellitzer G, Robine S (2011). Distinct ATOH1 and Neurog3 requirements define tuft cells as a new secretory cell type in the intestinal epithelium. J Cell Biol.

[13] Wang D, Dubois RN (2010). The role of COX-2 in intestinal inflammation and colorectal cancer. Oncogene.

[14] Nie D, Honn KV (2002). Cyclooxygenase, lipoxygenase and tumor angiogenesis. Cell Mol Life Sci.

[15] Tsujii M, Kawano S, DuBois RN (1997). Cyclooxygenase-2 expression in human colon cancer cells increases metastatic potential. Proc Natl Acad Sci.

[16] Fujita T, Matsui M, Takaku K, Uetake H, Ichikawa W, Taketo MM (1998). Size- and invasion-dependent increase in cyclooxygenase 2 levels in human colorectal carcinomas. Cancer Res.

[17] Marnett LJ (1992). Aspirin and the potential role of prostaglandins in colon cancer. Cancer Res.

[18] Eberhart CE, Coffey RJ, Radhika A, Giardiello FM, Ferrenbach S, Dubois RN (1994). Up-regulation of cyclooxygenase 2 gene expression in human colorectal adenomas and adenocarcinomas. Gastroenterology.

[19] Pugh S, Thomas GA (1994). Patients with adenomatous polyps and carcinomas have increased colonic mucosal prostaglandin E2. Gut.

[20] Shao J, Jung C, Liu C, Sheng H (2005). Prostaglandin E2 stimulates the beta-catenin/T cell factor-dependent transcription in colon cancer. J Biol Chem.

[21] Balch CM, Dougherty PA, Cloud GA, Tilden AB (1984). Prostaglandin E2-mediated suppression of cellular immunity in colon cancer patients. Surgery.

[22] Oshima M, Dinchuk JE, Kargman SL, Oshima H, Hancock B, Kwong E (1996). Suppression of intestinal polyposis in Apc delta716 knockout mice by inhibition of cyclooxygenase 2 (COX-2). Cell.

[23] Chulada PC, Thompson MB, Mahler JF, Doyle CM, Gaul BW, Lee C (2000). Genetic disruption of Ptgs-1, as well as of Ptgs-2, reduces intestinal tumorigenesis in min mice. Cancer Res.

[24] Smyth EM, Grosser T, Wang M, Yu Y, FitzGerald GA (2009). Prostanoids in health and disease. J Lipid Res.

[25] Patrono C, Patrignani P, García Rodríguez LA (2001). Cyclooxygenase-selective inhibition of prostanoid formation: transducing biochemical selectivity into clinical read-outs. J Clin Invest.

[26] Kang Y-J, Mbonye UR, DeLong CJ, Wada M, Smith WL (2007). Regulation of intracellular cyclooxygenase levels by gene transcription and protein degradation. Prog Lipid Res.

[27] Kirkby NS, Chan MV, Zaiss AK, Garcia-Vaz E, Jiao J, Berglund LM (2016). Systematic study of constitutive cyclooxygenase-2 expression: role of NF-κB and NFAT transcriptional pathways. Proc Natl Acad Sci U S A.

[28] Takeuchi K, Kita K, Hayashi S, Aihara E (2011). Regulatory mechanism of duodenal bicarbonate secretion: roles of endogenous prostaglandins and nitric oxide. Pharmacol Ther.

[29] Bjerknes M, Khandanpour C, Möröy T, Fujiyama T, Hoshino M, Klisch TJ (2012). Origin of the brush cell lineage in the mouse intestinal epithelium. Dev Biol.

[30] Schütz B, Jurastow I, Bader S, Ringer C, von Engelhardt J, Chubanov V (2015). Chemical coding and chemosensory properties of cholinergic brush cells in the mouse gastrointestinal and biliary tract. Front Physiol.

[31] von Moltke J, Ji M, Liang H-E, Locksley RM (2016). Tuft-cell-derived IL-25 regulates an intestinal ILC2-epithelial response circuit. Nature.

[32] Höfer D, Drenckhahn D (1992). Identification of brush cells in the alimentary and respiratory system by antibodies to villin and fimbrin. Histochemistry.

[33] Gerbe F, Sidot E, Smyth DJ, Ohmoto M, Matsumoto I, Dardalhon V (2016). Intestinal epithelial tuft cells initiate type 2 mucosal immunity to helminth parasites. Nature.

[34] Gerbe F, Legraverend C, Jay P (2012). The intestinal epithelium tuft cells: specification and function. Cell Mol Life Sci.

[35] Westphalen CB, Asfaha S, Hayakawa Y, Takemoto Y, Lukin DJ, Nuber AH (2014). Long-lived intestinal tuft cells serve as colon cancer-initiating cells. J Clin Invest.

[36] Powell DW, Mifflin RC, Valentich JD, Crowe SE, Saada JI, West AB (1999). Myofibroblasts. II. Intestinal subepithelial myofibroblasts. Am J Phys.

[37] Cohn SM, Schloemann S, Tessner T, Seibert K, Stenson WF (1997). Crypt stem cell survival in the mouse intestinal epithelium is regulated by prostaglandins synthesized through cyclooxygenase-1. J Clin Invest.

[38] Berschneider HM, Powell DW (1992). Fibroblasts modulate intestinal secretory responses to inflammatory mediators. J Clin Invest.

[39] Maskarinec G, Harmon BE, Little MA, Ollberding NJ, Kolonel LN, Henderson BE (2015). Excess body weight and colorectal cancer survival: the multiethnic cohort. Cancer Causes Control.

[40] Clauss W, Arnason SS, Munck BG, Skadhauge E (1984). Aldosterone-induced sodium transport in lower intestine. Effects of varying NaCl intake. Pflügers Arch Eur J Physiol.

[41] Geraedts MCP, Troost FJ, De Ridder RJ, Bodelier AGL, Masclee AAM, Saris WHM (2012). Validation of Ussing chamber technology to study satiety hormone release from human duodenal specimens. Obesity.

[42] Mahmood B, Matthiesen M, Damm B, Søren T, Jensen R, Balslev Backe M (2016). Phosphodiesterases in non-neoplastic appearing colonic mucosa from patients with colorectal neoplasia. BMC Cancer.

[43] Rothwell PM, Wilson M, Price JF, Belch JF, Meade TW, Mehta Z (2012). Effect of daily aspirin on risk of cancer metastasis: a study of incident cancers during randomised controlled trials. Lancet.

[44] Thun MJ, Jacobs EJ, Patrono C (2012). The role of aspirin in cancer prevention. Nat Rev Clin Oncol.

[45] Wang D, DuBois RN (2016). The role of prostaglandin E2 in tumor-associated immunosuppression. Trends Mol Med.

[46] Cuzick J, Thorat MA, Bosetti C, Brown PH, Burn J, Cook NR (2015). Estimates of benefits and harms of prophylactic use of aspirin in the general population. Ann Oncol.

[47] Koeberle A, Laufer SA, Werz O (2016). Design and development of microsomal prostaglandin E2 Synthase-1 inhibitors: challenges and future directions. J Med Chem.

[48] Nosho K, Sukawa Y, Adachi Y, Ito M, Mitsuhashi K, Kurihara H (2016). Association of Fusobacterium nucleatum with immunity and molecular alterations in colorectal cancer. World J Gastroenterol.

[49] Mima K, Ogino S, Nakagawa S, Sawayama H, Kinoshita K, Krashima R (2017). The role of intestinal bacteria in the development and progression of gastrointestinal tract neoplasms. Surg Oncol.

